# Henryk Nusbaum (1849–1937)

**DOI:** 10.1007/s00415-019-09528-1

**Published:** 2019-09-17

**Authors:** Jan Zamojski, Marcin Moskalewicz

**Affiliations:** Uniwersytet Medyczny Imienia Karola Marcinkowskiego W Poznaniu, Poznań, Poland

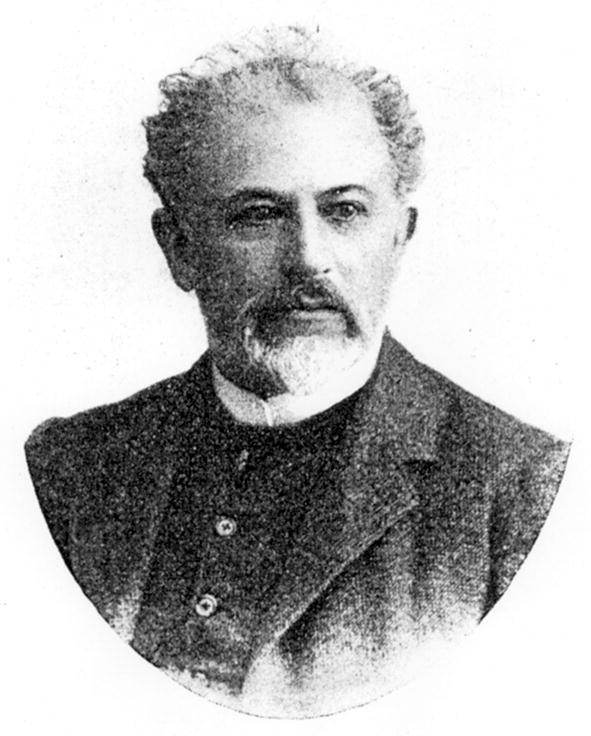



2019 marks the 170th anniversary of the birth of Henryk Nusbaum, a Jewish–Polish neurologist, physiologist, and philosopher of medicine. Experimenting on cats, Nusbaum showed the course of sympathetic nerve fibers in mammals connecting the stellate ganglion with cardiac muscle as well as the role of these fibers in increasing heart rate. He also demonstrated that these sympathetic nerve fibers are located in the vagus nerve above the middle cervical ganglion, and additionally the paralyzing action of curare on the parasympathetic fibers of this nerve [1, 2]. His scientific investigations also included the physiology of digestive and urinary systems. For instance, Nusbaum proved that detrusor muscle contractions can be stimulated by an impulse travelling from the brain via spinal and sympathetic nerves, and he described the latter’s course [3].

Nusbaum was born in Warsaw in a Jewish family practicing Reform Judaism, and his family had a great impact on his personal development [4]. He studied at the Medical Faculty of the Main School in Warsaw. Among his teachers were Henryk Hoyer and Feliks Nawrocki. After 1869, when the School was transformed into Imperial University of Warsaw, with Russian as the language of instruction, he attended the lectures of Tytus Chałubiński, whose ideas initiated the Polish School of Philosophy of Medicine. Nusbaum graduated in 1872 and from 1873 worked with Ignacy Baranowski on medical semiotics.

In 1874, he moved to the Imperial University of Dorpat (now Tartu, Estonia), where the language of instruction was German, and passed Examen rigorosum pro grado Doctoris medicinae. In 1875, Nusbaum defended his Ph.D. His opponents (“Ordentliche Opponenten”—a type of external examiners) were professors Ludwig Stieda, Alexander Schmidt and Rudolf Boehm [5]. Afterwards, he traveled abroad. In Vienna, he attended the lectures of neurologists Theodor Meynert and Moritz Benedikt, and in Paris, the lectures of Jean-Martin Charcot and the physiologist Alfred Vulpian.

Upon his return to Warsaw, Nusbaum began research at the physiology department and published works on the therapeutic aspects of electricity, the innervation of detrusor muscle, and the effects of poisons on submandibular glands [3, 6]. In 1882, he moved abroad again, this time to Bern, where he specialized in physiological chemistry under Marceli Nencki. Upon return, he applied to the Medical Faculty of the University of Warsaw for the academic degree of a docent in physiology (an equivalent of American associate professor). After he delivered two public lectures pro venia legendi, that is for an authorization to teach at a university, the faculty decided to bestow him the title. However, this decision was not approved by the tsarist authorities, officially due to his inadequate knowledge of Russian, a hardly believable reason. We may speculate that the true grounds had to do with Nusbaum being Jewish and with his family’s support for the 1863 January Uprising, a major failed Polish insurrection against the Russian Empire. In consequence, Nusbaum was forced to leave academia. Around 1883, being unable to head a hospital ward, he opened a private practice in neurology and internal medicine. His most famous patient was Eliza Orzeszkowa, nominated for the Nobel Prize in Literature in 1905. Nusbaum also played an important role in professional organizations and was one of the founding members of the Association of Polish Physicians.

Despite being pushed out of academia, Nusbaum continued pursuing his scientific studies. He gave a plenary lecture at the First Congress of Polish Neurologists, Psychiatrists, and Psychologists in Warsaw in 1909 entitled “W sprawie wskazań do stosowania narkotyków w cierpieniach układu nerwowego” (On indications regarding the use of drugs in the illnesses of the nervous system). His research in physiology and physiological chemistry explored, among others, the role of proteins in metabolism, the physiological effects of alcohol and other drugs, and the interaction of pancreas with selected poisons. Many of his papers were later republished in a large collection entitled “Writings in medical sciences. Physiology, Pathology, and General Therapy” [6]. Nusbaum also translated Sigismund Jaccoud’s [7] and Adolphe Combe’s [8] important works in pathology and neurology from French into Polish. While in private neurological practice, Nusbaum also advanced his interests in philosophy and ethics of medicine. These interests developed into a full-blown system [9]. His most influential work in this respect was “O wpływie czynności duchowych na sprawy chorobowe” (On the impact of spiritual activities on diseases), a paper first given at the 7th Congress of Polish Physicians and Natural Scientists in Lviv in 1894 [10].

After Poland regained its independence in 1918, Nusbaum’s scientific accomplishments were recognized by the medical faculty of the newly reformed University of Warsaw—in 1920, he was awarded the title of a docent (associate professor) in philosophy and logic of medicine (and not, as previously, in physiology), and 3 years later, the honorary title of a professor of philosophy of medicine. Nusbaum became the first scholar to teach philosophy of medicine as a subject on its own, and he published the first Polish academic textbook on this topic [11]. Today, Nusbaum is remembered as one of the leading characters of the Polish School of Philosophy of Medicine, a scientific and philosophical formation whose representatives were successful in combining scientific achievements with philosophical and bioethical reflection.
